# Clinical value of contrast-enhanced computed tomography (CECT) combined with contrast-enhanced ultrasound (CEUS) for characterization and diagnosis of small nodular lesions in liver

**DOI:** 10.12669/pjms.37.7.4306

**Published:** 2021

**Authors:** Jia-lian Liu, Dong Bao, Zong-li Xu, Xiang-ju Zhuge

**Affiliations:** 1 Jia-lian Liu, Department of Imaging, Linyi Central Hospital, Linyi, Shandong, 276400, P.R. China; 2 Dong Bao, Department of Imaging, Linyi Central Hospital, Linyi, Shandong, 276400, P.R. China; 3 Zong-li Xu, Department of Imaging, Linyi Central Hospital, Linyi, Shandong, 276400, P.R. China; 4 Xiang-ju Zhuge Department of Imaging, Linyi Maternal and Child Health Hospital, Linyi, Shandong, 276400, P.R. China

**Keywords:** Contrast-enhanced computed tomography, Ultrasound imaging, Small liver nodules, Degree of differentiation, Characterization and diagnosis

## Abstract

**Objectives:**

To explore the clinical value of contrast-enhanced computed tomography (CECT) combined with contrast-enhanced ultrasound (CEUS) for characterization and diagnosis of small nodular lesions in the liver and investigate the association between such small nodular lesions and the degree of tumor differentiation.

**Methods:**

Combined imaging modalities were performed on 120 patients who were admitted by Linyi Maternal and Child Health hospital from December 2018 to December 2020 and diagnosed with hepatic nodular lesions. The CT scans were interpreted by two senior imageologists while the ultrasound scans were analyzed by two senior sonographers. A comparative analysis was carried out on different scan modes and the postoperative or post-puncture pathological results using the t-test, the χ2 test, and the Pearson’s correlation analysis.

**Results:**

Compared to the pathological results, definite diagnoses of 55 malignant cases were made using CECT alone, with the coincidence rate of 78.6%; CECT combined with CEUS formed correct diagnoses in 64 cases, and the coincidence rate was up to 91.4%. The difference between the two scan modes was statistically significant (p= 0.03). Based on pathological diagnosis, seventy out of the 120 cases of small nodular lesions were identified as malignant, while the other 50 cases were benign. The single imaging modality diagnosed 63 malignant and 57 benign nodules, whereas the combined modalities identified 68 malignancies and 52 benign conditions. Compared to CECT as a single imaging modality, the combined modalities showed a higher degree of sensitivity and accuracy, and the difference was statistically significant (sensitivity: p= 0.03; accuracy: p= 0.02); in the malignant cases, the magnitudes of contrast enhancement of CT and ultrasound imaging decreased with an increase in the degree of differentiation, indicating a negative correlation between these factors.

**Conclusions:**

CECT combined with CEUS has a higher coincidence rate, greater sensitivity, and better diagnostic accuracy when being used for characterization and diagnosis of small nodular lesions in the liver. A higher degree of tumor differentiation means a decreased magnitude of contrast enhancement and a blurrier boundary, which indicates that CECT and CEUS are complementary to each other in classifying malignant liver nodules. The use of the combined imaging modalities shows clinical value for characterizing small liver nodules and predicting the degree of malignancy.

## INTRODUCTION

Liver cancer is a commonly seen gastrointestinal malignancy. With a higher degree of malignancy indicating an increased risk of death, the disease can be a life-threatening challenge.^1^ About 80% of hepatocellular carcinoma (HCC) cases occur after chronic hepatitis and cirrhosis.^2^ With an insidious onset and a lack of characteristic clinical signs and symptoms, it is relatively difficult to diagnose liver cancer at the very early stage.^3^ Early characterization and diagnosis of liver cancer have clinical significance for choosing treatment options and predicting prognosis. Generally, CT scans of the liver are blurred by the abundant blood supply, and there is noise interference. Reportedly, the sensitivity of CT in the detection of liver nodules, especially the smaller ones, is relatively low.^4^ Therefore, Other complementary methods are needed to improve the detection rate.^5^ CEUS surveillance is a safe, cost-effective, and highly accurate imaging modality widely used for the detection of malignant focal liver lesions based on exclusive ultrasonic sequences and FDA-approved microbubbles. CEUS enables real-time representation of dynamic events, making it an ideal complement to CT and MRI for the characterization of indeterminate lesions.^6^ The use of CECT combined with CEUS has clinical value in that it supports the characterization and diagnosis of small nodular lesions in the liver and improves prediction of malignant lesions. Details are reported as follows:

## METHODS

### Ethical approval

The study was approved by the Institutional Ethics Committee of Linyi Maternal and Child Health Hospital (No.LCH20210202058), and written informed consent was obtained from all participants.

### Inclusion criteria

(1) Imageology indicated small nodular lesions (≤ 5 cm) in the liver;^7^ (2) Definitive diagnosis was given after operation or biopsy, and pathological results were available;^8^ (3) CECT and CEUS surveillance were performed within a month (30 days) before operation or biopsy; (4) No multiple but solitary lesion was detected; (5) Complete clinical materials were available; (6) The patient, with clear consciousness, had no history of mental disorder; (7) The patient and his/her family agreed to this study, and the patient was cooperative and compliant throughout the treatment course.

### Exclusion criteria

(1) It was not the first time for the patient to seek medical attention because of the liver condition; (2) Tumors were detected in the liver and another part of the body; (3) The patient had severe primary comorbidities in other tissues, accompanied by serious pleural effusion and ascites and jaundice; (4) Clinical or pathological materials were incomplete; (5) The patient was not able to participate in the study independently because of mental or cognitive disorder; (6) Multiple lesions were detected.

This study included 120 patients who were admitted by Linyi Maternal and Child Health hospital from December 2018 to December 2020 and diagnosed with hepatic nodular lesions. These patients were divided into a malignant group and a benign group by their pathological results. The malignant group was formed by 70 patients, including 43 males and 27 females aged between 54 and 71 (mean age: 62.81±6.72, max. nodule diameter: 3.31±1.20 cm, course of disease: 1.31±0.55 yrs). In the benign group, there were 31 males and 19 females at the age between 52 and 70 (mean age: 61.39±7.35, max. nodule diameter: 2.83±1.04 cm, course of disease: 2.06±0.49 yrs). There was no statistically significant difference between the two groups in sex, age, and other demographic data, indicating intergroup comparability in these aspects (Table-I). Nodular lesions in the malignant group had a diameter larger than the benign ones, and the difference was statistically significant (p= 0.02). Compared with the benign group, the malignant group had a shorter course of disease, and the difference had statistical significance (p= 0.00). In terms of pathological results, the malignant group was comprised of HCC (n= 58), cholangiocellular carcinoma (CCC, n= 7), and mixed tumors (n= 5); the benign group consisted of focal nodular hyperplasia (FNH, n= 32), hepatic adenoma (HA, n= 9), hepatic cystadenoma (HCA, n= 6), and chronic granulomatous disease (CGD, n= 3).

**Table I:** Malignant group vs. benign group: demographic data (X¯ ±S).

*Indicator*	*Malignant Group (n= 70)*	*Benign Group (n= 50)*	*t/χ^2^*	*p*
Age*	62.81±6.72	61.39±7.35	0.20	0.27
Male (n/%)*	43 (61%)	31 (62%)	0.00	0.95
Nodule diameter (cm)Δ	3.31±1.20	2.83±1.04	2.30	0.02
Course of disease (yrs)Δ	1.31±0.55	2.06±0.49	7.70	0.00

* p>0.05; Δ<0.05.

The patient who refrained from eating as required was placed in a supine position for routine plain CT scanning using a Philips 64-slice helical CT scanner, with the slice thickness of 4 mm and the slice gap of 5 mm. After the plain CT scanning, 80 mL of iohexol was injected into the ulnar vein with a high-pressure syringe at the flow rate of 3-4 ml/s. Arterial-phase CT imaging started 24-26 s after the injection, followed by portal venous- and late washout-phase imaging (45-60 s and 120-180 s delay, respectively). The degree of contrast enhancement of the lesion was observed.

### Ultrasound microvascular imaging (UMI)

The patient was asked to fast several hours prior to scanning, and a 3-5 MHz Philips color doppler ultrasound scanner with a convex array probe was used for UMI with the patient lying in a supine position. Before the procedure, the patient and his/her family were informed of possible adverse reactions to UMI. First of all, routine ultrasound imaging was performed to read the location, shape, and size of the nodular lesion. The target nodule was subject to ultrasound imaging surveillance. Bracco Sono Vuc (a contrast agent) was mixed with 5 mL normal saline to prepare microbubble suspension.^9^ The contrast agent (2 mL) was administrated with ulnar vein injection, followed by washing with 5.0 mL of normal saline. The patient was asked to control his/her breath before setting the timer for 60 s, during which period, real-time scanning of perfusion occurred. This process was divided into three phases, including the arterial phase (10-30 s), the portal venous phase (30-120 s), and the late washout-phase (180-360 s). Real-time ultrasound imaging was useful to observe the features of a tumor. The degree of contrast enhancement was classified as low, medium, and high.^10^ All test results were assessed by two senior imageologists and two senior sonographers.

### Outcome measures

(1) Calculating the coincidence rates of different imaging modalities in relation to the pathological results; (2) Comparing the sensitivity, specificity, and accuracy of the single and combined imaging modalities for the characterization of small nodular lesions in the liver, with the pathological results as the standard of diagnosis; (3) Analyzing the correlations between liver nodules at a varying degree of malignancy and the results produced by the combined imaging modalities.

### Statistical Analysis

Statistical analysis was performed using SPSS 20.0, with the measurement data being represented by (X¯ ±S). Intergroup comparison was examined with the independent-samples t-test, and the comparison of rates was examined with the χ^2^ test. Correlations were expressed by Pearson’s correlation coefficients. P< 0.05 was considered statistically significant.

## RESULTS

Comparing the detection rates of the single and combined imaging modalities to the pathological results (Table-II), CECT alone identified 55 malignant nodules, and the coincidence rate was 78.6% (55/70); the combined imaging modalities formed definite diagnosis of 64 malignant cases, and the coincidence rate was 91.4% (64/70). There is a significant difference between these techniques (p= 0.03).

**Table II:** Coincidence rates of different imaging modalities in relation to pathological results (X¯ ±S), n=70.

*Group*	*Malignant Cases*	*Malignant Cases Confirmed by Pathological Diagnosis*	Coincidence Rate*
CECT	55	70	78.6%
CECT combined with CEUS	64	70	91.4%
χ^2^			4.53
p			0.03

* p<0.05.

According to the pathological results, there were 70 malignant cases and 50 benign cases. When different imaging modalities were used for characterization and diagnosis, sixty-three malignancies and 57 benign nodules were detected using CECT alone, whereas 68 malignancies and 52 benign cases were identified with the combined imaging modalities. The combined imaging modalities outperformed the single imaging modality in screening sensitivity and diagnostic accuracy, and the difference was statistically significant (sensitivity: p= 0.03; accuracy: p= 0.02). Tables-III & IV.

**Table III:** Correlation analysis of malignant and benign tumors identified by the single and combined imaging modalities in relation to the pathological results.

*Pathological Results*	*CECT*			*CECT combined with CEUS*		

	*Malignant Cases*	*Benign Cases*	*Total*	*Malignant Cases*	*Benign Cases*	*Total*
Malignant Cases	55	15	70	64	6	70
Benign Cases	8	42	50	4	46	50
Total	63	57	120	68	52	120

**Table IV:** Diagnostic sensitivity, specificity, and accuracy of the single and combined imaging modalities.

*Group*	Sensitivity*	*Specificity*	Accuracy*
CECT	(55/70)	88.33% (110/120)	69.17% (97/120)
CECT combined with CEUS	(64/70)	91.42% (46/50)	88.33% (110/120)
χ^2^	4.53	0.52	5.94
p	0.03	0.21	0.02

* p<0.05.

Correlation analysis showed that the degrees of contrast enhancement of CT and UMI were reduced as the differentiation degree of a malignant liver nodule increased, indicating a negative correlation between the degree of contrast enhancement of CT and UMI and the degree of tumor differentiation (Table-V). In other words, CT and UMI are two complementary modalities for the determination of differentiation degrees of malignant liver nodules.

**Table V:** Correlations between different pathological patterns and UMI results.

*Differentiation Degree*	*CECT*	*UMI*
Low differentiation	-0.37	-0.41
Medium differentiation	-0.32	-0.36
High differentiation	-0.28	-0.22

## DISCUSSION

Cirrhotic nodules and HCC are two types of space-occupying liver lesions commonly seen in clinical practice.^11^ HCC mostly occurs after chronic hepatitis and cirrhosis. It is difficult to make an early diagnosis of HCC because the disease has no specific clinical signs and symptoms in early stages. For space-occupying liver lesions, accurate diagnosis and timely intervention during the early onset are critical to improve the prognosis and quality of life.^12^ In clinical practice, despite the detection of alpha-fetoprotein (AFP) and other tumor markers,^13^ imageology still plays a fundamental role in deciding treatment regimens and predicting prognosis.^14^

CECT as an imaging technique is based on the theory of hepatic arterial blood supply to the focal liver lesion.^15^ With tissue or vascular specific contrast agents, CT has become a powerful tool to monitor tumor growth in animal livers.^16^ CECT is able to grasp the “fast-in and out” feature of HCC lesions and exhibit the blood flow in the tumor.^17^

A normal liver derives 80% of its blood from the portal vein and the other 20% from the hepatic artery. CECT is characterized by efficient scanning and high diagnostic accuracy as it supports total liver scanning during the arterial and portal venous phases, respectively.^18^ However, in clinical practice, the surrounding blood flow may interfere with the detection of smaller nodules or focal liver lesions. Besides, it is sometimes difficult to distinguish benign lesions from HCC lesions. All this has imposed a radical challenge to the characterization and diagnosis of HCC. CT and MRI occasionally fail in characterizing indeterminate liver lesions.

As a unique imaging modality that supports purely intravascular contrast agents and enables real-time evaluation of contrast enhancement, CEUS is an ideal complement to CT or MRI for the characterization of indeterminate liver lesions.^19^ Ultrasound imaging has recently obtained approval of the U.S. Food and Drug Administration (FDA) for detection of liver injury. Zarzour et al.^20^ pointed out that ultrasound imaging had clinical value in distinguishing benign liver lesions from malignant ones. Ultrasound imaging uses inert gases as contrast agents and depicts the histological features of the liver based on the blood perfusion of liver nodules and the acoustic differences between the liver nodules and the normal liver parenchyma.^21^ A clinical research discovered that most cirrhotic nodules convert to high-grade dysplastic nodules by forming low-grade dysplastic nodules (LGDNs) through nodular regeneration, and eventually develop into early HCCs; therefore, hepatocellular development is considered part of the process of angiogenesis and vascularization.^22^ Tumor angiogenesis is characterized by unpaired arteries and sinusoidal capillarization,^23^ which provides a theoretical basis for the characterization of relevant vascular patterns on suspicious sites. As a radiation-free and cost-effective imaging modality that enables real-time monitoring, CEUS has been extensively used in the diagnosis of liver cancer.^24^ However, unlike other types of liver cancer, cirrhosis causes substantial damage to the liver structure. Moreover, it is difficult to distinguish cirrhotic nodules from space-occupying lesions associated with early-stage liver cancer. Therefore, CEUS is viewed as a favorable complement to CT or MRI for the diagnosis of HCC in a cirrhotic liver.^25^

This study revealed that using CECT alone could yield a coincidence rate of 78.6% whereas CECT combined with CEUS had a coincidence rate of 91.4%, and the difference was statistically significant (p= 0.03); According to the pathological results, 70 out of the 120 patients had malignant focal liver lesions and the rest 50 patients were diagnosed with benign nodules. Compared to CECT as a single imaging modality, the combined modalities showed a higher degree of sensitivity and accuracy, and the difference was statistically significant (sensitivity: p= 0.03; accuracy: p= 0.02); in the malignant cases, the magnitudes of contrast enhancement of CT and ultrasound imaging decreased with an increase in the degree of differentiation, indicating a negative correlation between these factors.

### Limitation of the study

This study only included a relatively modest number of cases; according to the pathological results, most of the malignant nodules were HCCs, and thus there was a very limited proportion of other pathological patterns. More cases should be included to expand the sample size, thereby further studying the clinical value of using CECT combined with CEUS for characterization and diagnosis of malignant nodules with different pathological patterns. Additionally, CEUS is still in the early stage of clinical application, and the performance of ultrasound imaging can be affected by many factors. To produce a highly objective and scientific research, further improvements will be made, such as expanding the sample size, and discussing different pathological patterns in this study.

## CONCLUSION

The combined imaging modalities not only have a higher coincidence rate when being used for the characterization of small liver nodules, but also surpass the single imaging modality in screening sensitivity and diagnostic accuracy. CECT and CEUS are complementary to each other in determining the degree of differentiation of malignant liver nodules. The use of CECT combined with CEUS shows clinical value for characterizing small liver nodules and predicting the degree of malignancy.

### Authors’ Contributions:

**JLL &** **XJZ:** Designed this study and prepared this manuscript, and are responsible and accountable for the accuracy or integrity of the work.

**DB:** Collected and analyzed clinical data.

**ZLX:** Significantly revised this manuscript.
